# Biomimetic Enamel-like Crystals: A Versatile Platform for Unraveling the Basic Mechanisms of Demineralization and Remineralization

**DOI:** 10.1021/acsami.5c13544

**Published:** 2025-08-28

**Authors:** Jinke Chang, Mahdi Tavakol, Cyril Besnard, Alexander M. Korsunsky, Jin-Chong Tan

**Affiliations:** Department of Engineering Science, 6396 University of Oxford, Oxford, Oxfordshire OX1 3PJ, U.K.

**Keywords:** human enamel, hydroxyapatite, fluoridized hydroxyapatite, remineralization, demineralization, in situ observation

## Abstract

Enamel is a cellular, nonregenerative, highly mineralized tissue essential for the mechanical durability and wear resistance of human teeth. Combating its degradation necessitates effective remineralization strategies, with hydroxyapatite (HAp) playing a central role in both natural and synthetic enamel restoration. Fluoride incorporation enhances HAp stability, forming fluoridated hydroxyapatite (FHAp), which is widely used to prevent or resist dental caries and improve remineralization. However, a mechanistic understanding of demineralization and remineralization remains incomplete due to the limitations of conventional ex situ techniques, which fail to capture real-time crystal dissolution and growth dynamics. In this study, we developed and applied a facile synthesis method for oriented FHAp nanocrystals under ambient pressure and at body temperature. This unlocks the possibility of direct in situ liquid imaging using atomic force microscopy (AFM) that serves as a platform for direct observation of demineralization and remineralization processes at the nanoscale. Investigation of the morphology, spectroscopy, and mechanical properties of nanocrystals grown in different conditions elucidated the effect of the substitution rate of fluorine through both in situ and ex situ studies. The findings presented offer a generic approach for understanding the re/demineralization mechanisms in enamel and demonstrate the potential for charting biomimetic enamel restoration pathways.

## Graphical abstract

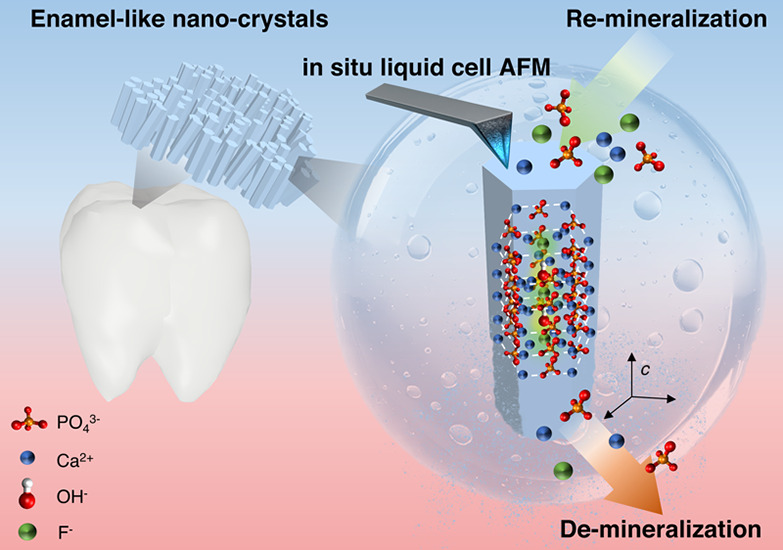

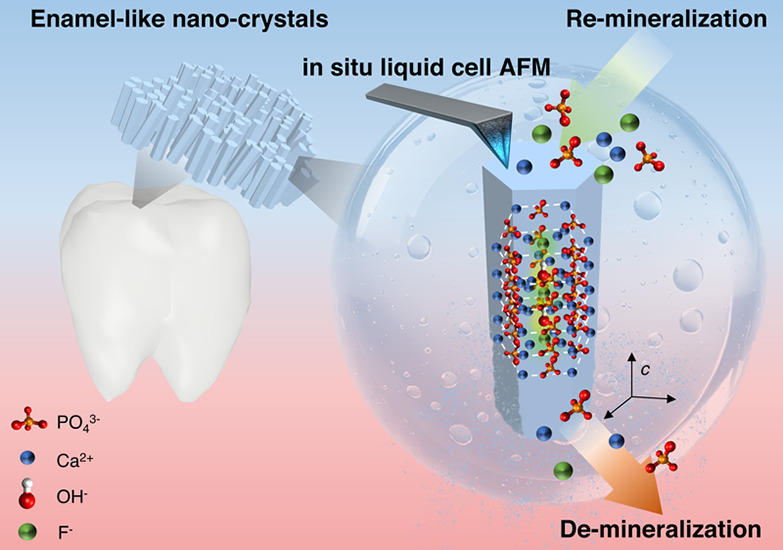

## 1 Introduction

Dental enamel plays a fundamental role in human daily life, providing the mechanical strength and wear resistance of teeth to withstand significant thermomechanical loads during chewing and biting.^1,2^ However, mature human enamel is nonregenerative, making its degradation and loss a critical concern in healthcare, restorative dentistry and biomedical materials research.^3^ Damage to enamel compromises chewing ability and overall oral health, necessitating the search for effective strategies for enamel remineralisation and protection.^4^ The primary inorganic component of enamel, hydroxyapatite Ca_10_(PO_4_)_6_(OH)_2_, commonly referred to as HAp, is central to both natural remineralisation processes and synthetic enamel restoration approaches.^5^

Recent efforts have focused on developing new remineralisation techniques using hydroxyapatite-based materials, aiming to enhance the mechanical durability and acid resistance.^6,7^ Enamel-like nanostructures have also been widely explored to rebuild the hierarchical architecture of natural enamel.^8^ In most enamel remineralisation processes, calcium-deficient hydroxyapatite is formed, characterized by a Ca/P ratio that is lower than the stoichiometric value of HAp (Ca/P = 1.67). Moreover, restored enamel frequently incorporates ions such as Sr^2–^, Zn^2–^, Mg^2–^, CO_3_^2–^, and F^–^, all of which influence the chemical and mechanical properties of the demineralised and remineralised crystals.^9–11^ The properties of these newly formed crystals are inherently linked to their chemical composition, morphology, and nanostructure. Among various remineralisation agents, fluoride is the most widely used additive in toothpastes and dental treatments due to its effectiveness in enhancing tooth remineralisation.^12,13^ By substituting hydroxyl groups (OH^–^) in the hydroxyapatite lattice, fluoride promotes the formation of fluoridated hydroxyapatite, Ca_10_(PO_4_)_6_(OH)_2–*x*_F_*x*_, also referred to as FHAp.^14^ Fluoride substitution improves crystallinity, reduces solubility, and enhances acid resistance, making FHAp a promising candidate for a synthetic enamel-like material.^15^ Fluoride incorporation further influences crystal morphology,^16^ growth kinetics,^17^ surface charge and energy states,^18^ contributing to improved chemical and mechanical stability.^19^ Another important aspect in the nanoscale mechanical and chemical property characterization of enamel-like HAp crystals concerns their anisotropy with respect to crystal planes and orientations: specifically, the basal plane with a {001} normal (aligned with the *c*-axis) and the prismatic planes with {100}-type normals.^20^ It has been reported in the literature that in HAp the *c*-axis direction corresponds to the highest values of hardness and mechanical stiffness, but simultaneously shows the lowest chemical resistance to acidic dissolution.^21^ In the natural hierarchical design of HAp-rich dental enamel, the *c*-axis orientation dominates in the direction of the occlusal pressure, ensuring maximum contact durability under high loads.^22,23^ However, this orientation also predetermines high susceptibility to the attack from the acidic metabolic products of bacterial metabolism. These structural and chemical characteristics are critical for designing biomimetic enamel replacements that effectively resist demineralisation and support remineralisation. Nonetheless, the mechanistic understanding of these processes remains insufficient which requires further observation and analysis.

Advancements in nanoscale characterization techniques have significantly enhanced the understanding of enamel demineralisation and remineralisation mechanisms.^20,24,25^ For example, recent developments in transmission electron microscopy (TEM) have been employed to examine crystal lattice distortions, defects, and ion-substituted apatite structures, providing high-resolution imaging of structural transformations in biomimetic HAp.^26–28^ Moreover, atomic force microscopy, particularly in liquid environments, has been developed to capture real-time surface dynamics at the nanoscale, revealing nucleation events, crystal growth kinetics, and dissolution patterns.^29,30^ AFM-based force spectroscopy further enables the quantification of local mechanical properties, offering insights into the evolution of nanomechanical stability during mineralization.^31^ Despite these advancements, a major limitation persists: many existing studies rely on synthetic enamel-like HAp produced via hydrothermal or other chemically intensive processes.^32^ These methods typically require high temperatures, elevated pressures, or nonphysiological reaction conditions,^33^ deviating significantly from the requirements for many in situ characterization techniques. Furthermore, while ex-situ microscopic and spectroscopic analyses provide valuable compositional and morphological insights, they fail to capture the dynamic processes of crystal dissolution and growth in real-time. The lack of a precisely controlled, physiologically relevant platform for in situ characterization limits the ability to correlate nanoscale structural evolution with chemical interactions during remineralisation.

In this work, we address the challenges by employing in situ liquid cell atomic force microscopy (LC-AFM) to study the real-time remineralisation and demineralisation processes of enamel-like oriented FHAp crystals. The chemical and mechanical properties of these crystals are systematically examined, allowing us to explore their performance in terms of structural stability and acid resistance. The approach offers a facile and versatile basis for investigating enamel-mimicking materials, bridging the gap from ex-situ characterization to dynamic in situ studies. The findings offer mechanistic insight into the prevalence of caries in modern populations with sugar-rich diets and may suggest the future research directions for optimal remineralisation strategies.

## 2 Results and Discussion

### 2.1 Controlled Orientation and Morphology of Fluoridated Hydroxyapatite

Various methods have been developed for the fabrication of enamel-like hydroxyapatite crystal arrays, including layer-by-layer deposition,^34^ rotary evaporation techniques,^35^ and bilayer hydrogels.^36^ These approaches have shown promise in mimicking the hierarchical structure of enamel. Additionally, hydrothermal synthesis is widely recognized as a means of producing highly crystalline HAp nanorod arrays. The success of these methods underscores the importance of precisely controlling the nucleation and growth kinetics of calcium phosphate crystals, as well as their morphology, on various substrates. Despite these advancements, there remains a pressing need for the development of in situ fabrication techniques that can operate under ambient or physiological conditions, which are critical for real-world applications.

Fluoride ions (F^–^) have been found effective in promoting the growth of densely packed apatite crystals. Research has demonstrated that the substitution of hydroxyl groups with fluoride ions within the crystal lattice can regulate the crystal morphology.^37^ Building on this principle, the present study proposes a strategy for the growth of hydroxyapatite crystals at body temperature (37 °C). Amorphous calcium phosphate (ACP) in the form of compressed pellets offers a convenient substrate and source of building material (calcium and phosphate sources) for enamel-like HAp synthesis.^38,39^ The ACP substrate was immersed in a growth buffer solution, forming a crystalline overgrowth layer on its surface (see Section 4). By meticulously controlling the fluoride concentration in a buffer solution with Ca^2+^ and PO_4_^3–^, the method allowed the synthesis of FHAp crystals with tunable crystal structure and morphology. More importantly, the strategy was found to be specifically suitable for in situ observation of remineralisation and demineralisation processes under ambient pressure and at moderate temperature.

Figure 1a–c schematically illustrates the advantage of using the present strategy as a facile platform for studying the crystallization, morphology, mechanical and chemical properties of fluoridated apatite crystals in the re/demineralisation process. The controlled synthesis process allows the morphology, orientation, and size of the FHAp crystals to be finely tuned. Under the conditions with higher fluoride concentrations (1.5 mM), FHAp crystals adopt a highly faceted and well-oriented hexagonal rod-like morphology (Figure 2a,b). In contrast, fluoride-deficient (0.5 mM) conditions resulted in the formation of ovoid-shaped FHAp crystals, revealed by AFM topographic imaging in air as shown in Figure 2c,d. The well-ordered structure underscores the role of fluoride substitution in facilitating the crystallographic alignment and lattice densification, thereby enhancing crystallinity and structural stability. However, beyond the observed differences in crystal morphology, a deeper understanding of the mineralization process, crystal composition, and the properties of the nanocrystals is required to elucidate fully their growth mechanisms and functional characteristics.

**Figure 1:**
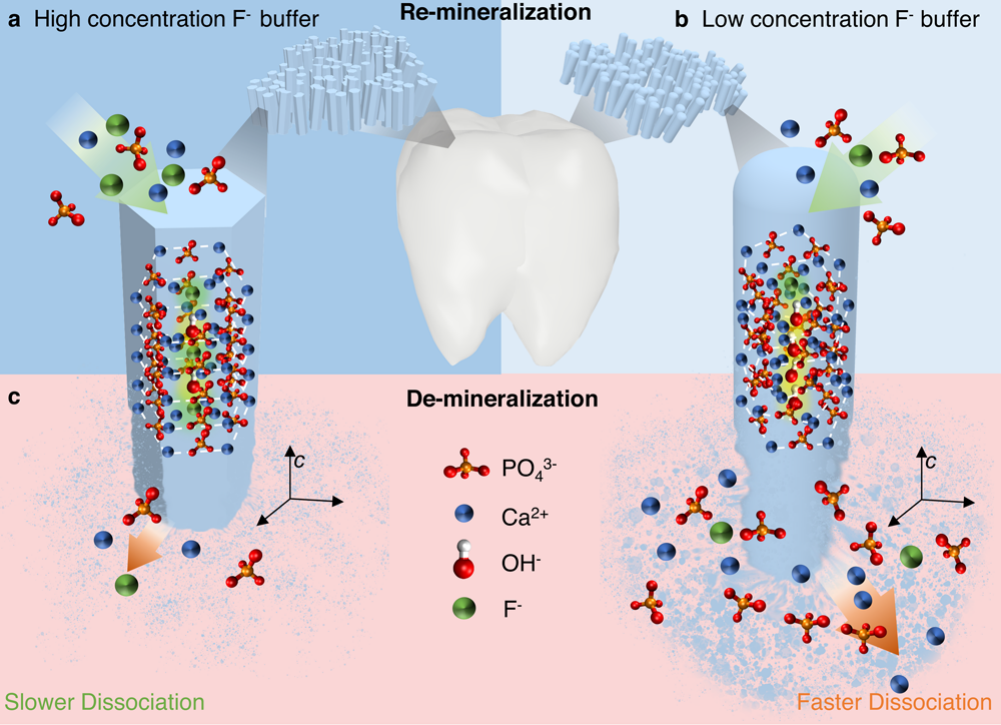
Schematic illustration of the remineralization and demineralization of oriented fluoridated hydroxyapatite with different fluorine substitutions. (a) Highly oriented hexagonal rod-shaped FHAp crystals formed in a higher concentration fluoride buffer condition (1.5 mM). (b) Ovoid-shaped crystals formed at a lower concentration of fluoride buffer (0.5 mM). (c) In situ demineralization observation reveals the dissociation rate of the crystals in acid and allows the assessment of chemical stability.

**Figure 2:**
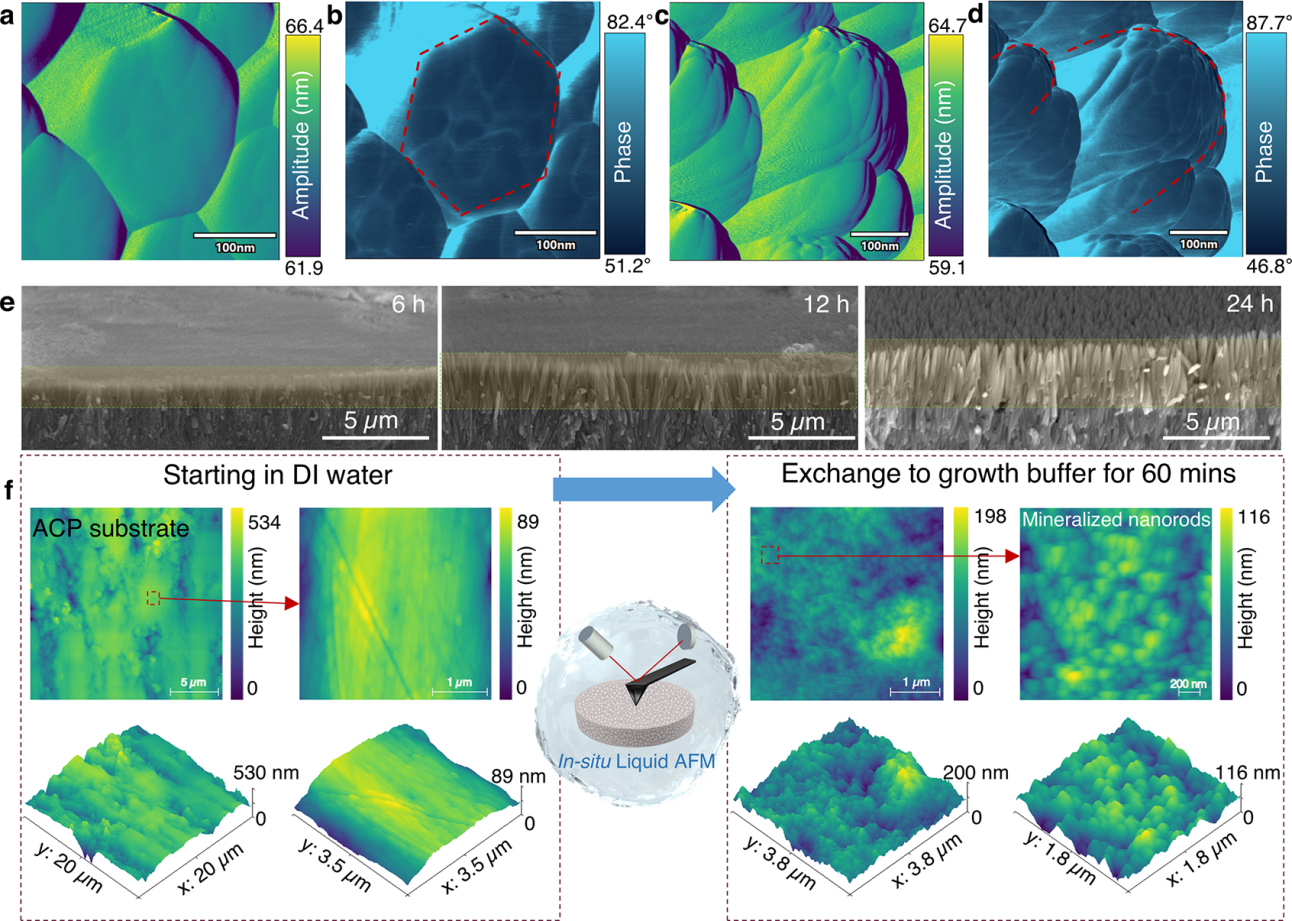
In situ and ex situ observation of the mineralization process on ACP substrates for tunable morphology and orientation. (a) AFM height topography image of hexagonal FHAp crystals. (b) AFM phase image of the hexagonal rod-like FHAp crystal. (c) AFM height image of the ovoid-shaped FHAp crystals. (d) AFM phase image of a single hexagonal FHAp crystal. (Imaging with a sharp probe with tip radius <5 nm, NuNano Scout 350) (e) Representative scanning electron microscopy (SEM) images of FHAp crystals grown in 0.5 mM fluorine buffer for 6, 12, and 24 h. (f) In situ liquid AFM observation of the rapid mineralization process in the 0.5 mM fluorine growth buffer solution over 60 min.

The microstructure resulting from time-dependent oriented crystal growth was characterized ex situ using SEM and powder X-ray diffraction (XRD). ACP substrates were immersed in the growth buffer (F^–^ molar ratio of 0.5 mM) and collected at 0.5, 1, 3, 6, 12, and 24 h for further characterization. Representative cross sections of the samples are displayed in Figure 2e. The upper crystalline layers are highlighted which grows from 0.47 ± 0.08 μm at 6 h to about 1.62 ± 0.24 μm by 12 h and 2.70 ± 0.29 μm by 24 h. The structures of ovoid-shaped oriented crystal layer were in agreement with the research by Onuma and Iijima^38^ and Carella et al.^37^ Namely, the XRD patterns reveal the gradual development of well-defined crystalline features, with two sharp peaks emerging at 2θ = 26.1 and 53.4°, indicative of increasing crystallinity and preferred orientation (Figure S1a). The reference XRD pattern, based on the standard hydroxyapatite database reford (JCPDS File No. 09-0432), assumes perfect alignment along the crystallographic [001] direction and show characteristic reflections at 25.7° (002) and 53.1° (004). These reflections correspond to lattice planes perpendicular to the *c*-axis, indicating preferential exposure of the {001} basal plane. The strong agreement between the experimental and standard XRD patterns confirms that the crystals grow predominantly with their *c*-axis oriented normal to the substrate. To quantify this anisotropic alignment further, the intensity ratio of the (002) to (112) reflections was analyzed. At 0.5 h of immersion, the (002)/(112) intensity ratio was 0.75, suggesting near-random orientation. This ratio increased significantly to 109.5 after 24 h, demonstrating a pronounced preferential growth along the *c*-axis as crystallization progressed (Figure S1b).

The Lotgering orientation factor (*L*) was used to quantify the degree of alignment along the [00*l*] direction. This factor indicates the orientation degree of crystalline nanorod arrays, where their long axis (parallel to the crystallographic *c*-axis) is perpendicular to the substrate.^40^ An *L* value close to 1 indicates perfect alignment, while a value close to 0 suggests random orientation. The Lotgering factor exhibited a rapid increase within the first 6 h, reaching 0.756, and further increased to 0.954 by 24 h, confirming the progressive enhancement of anisotropic alignment (Figure S1c). XRD patterns of both hexagonal rod-like and ovoid crystals after 24 h of growth were dominated by the (002) and (004) reflections, indicative of strong *c*-axis orientation along the basal planes. These strong reflections overshadow the weaker peaks, which are not sufficiently pronounced to allow for meaningful comparison between samples. To facilitate comparison between FHAp and HAp, XRD data collected at 12 h, where peak dominance is reduced, are presented in Figure S1d. The characteristic reflections observed at 25.9° (002), 31.8° (211), 34.0° (300), and 53.2° (004) align well with both the commercial HAp standard and the JCPDS reference card (File No. 09-0432), indicating consistent crystallographic structure. No significant peak shifts were detected, suggesting that fluoride incorporation at this stage does not result in measurable lattice distortion. Time-resolved XRD data show that crystallization begins within the first 30 min, characterized by rapid aggregation of precursor phases. During this initial period, the crystal orientation is largely disordered. As mineralization proceeds, alignment along the [00*l*] direction becomes increasingly pronounced. These results point to a progressive structural ordering process, which may benefit from further validation using in situ characterization techniques.

The dynamic process of mineralization on the ACP substrate was further observed using an in situ liquid LC-AFM as demonstrated in Figure 2f and Supplementary Video 1. Approximately 100 μL of deionized (DI) water was used to immerse the ACP substrate and AFM probe (FS-1500) for an initial stabilization scan lasting ∼10 min. The DI water was then slowly exchanged with the growth buffer using a continuous injection system, marking the initiation time point of FHAp crystal growth. The dynamic observation was maintained for approximately 60 min. As shown in Figure 2f, time-resolved AFM imaging reveals the progressive nucleation and growth of mineral clusters, in agreement with the trends inferred from the time-resolved XRD data in Figure S1. The real-time video suggests that mineral deposition occurs in a layer-by-layer manner, initially concentrating in nanoscopic concave regions of the substrate. Individual clusters subsequently coalesce and integrate into continuous mineralized regions. As these low-lying areas become filled and mineral clusters accumulate on the surface, lateral growth along the prismatic plane slows down, eventually leading to vertical (*c*-axis) competition for free space. This competition directs the height-oriented growth of FHAp crystals, promoting a high degree of anisotropy in the crystal development on the surface of the ACP substrate. It is worth noting that video-speed LCAFM (>20 Hz scanning frequency) introduced some disturbance at the crystal–liquid interface, likely due to high-frequency probe vibrations interfering with ion exchange processes. Long-term tracking of a single crystal over 1 h proved extremely challenging, as significant height changes along the *c*-axis often caused the probe to lose tracking with the surface. This required repeated readjustment of operational parameters, resulting in the loss of the observation window.

### 2.2 Chemical Composition and Molecular Structure of Fluoridated Hydroxyapatite

Figure 3 highlights the effect of fluoride concentration on the morphology and composition of FHAp crystals. At higher fluoride molar ratios, FHAp forms well-defined faceted hexagonal crystals, as shown in the SEM image (Figure 3a). The corresponding energy dispersive X-ray spectroscopy (EDS) elemental mapping (Figure 3b) and distribution maps (Figure 3c) confirm the uniform incorporation and distribution of phosphorus (P), oxygen (O), calcium (Ca), and fluoride (F) across the crystal population. EDS analysis of these crystals (Figure 3g) reveals the elemental composition: O (39.3 wt %), Ca (34.0 wt %), P (17.3 wt %), and F (4.5 wt %). The calculated atomic Ca/P ratio is approximately 1.52, slightly below the stoichiometric value of hydroxyapatite (1.67), reflecting the influence of fluoride substitution. The F:Ca atomic ratio was calculated as 0.279, which exceeds the theoretical ratio of 0.2 for a fully substituted FHAp (Ca_10_(PO_4_)_6_F_2_). While this could indicate a fluoride-rich, nonstoichiometric FHAp structure, it is also possible that residual, unincorporated fluoride remains adsorbed on the crystal surface or is trapped within grain boundaries, contributing to an overestimation of fluoride content in EDS analysis. The presence of oxygen (39.3 wt %) and phosphorus (17.3 wt %) suggests that secondary fluoride phases are unlikely, although further verification by spectroscopy would be necessary. In contrast, FHAp synthesized at lower fluoride concentrations exhibits an ovoid-shaped morphology (Figure 3d). EDS mapping (Figure 3e,f) reveals the distribution of P, O, Ca, and F in these crystals, indicating lesser fluoride incorporation. EDS analysis (Figure 3h) shows an elemental composition of O (35.6 wt %), Ca (34.1 wt %), P (18.1 wt %), and F (2.7 wt %), yielding a Ca/P atomic ratio of 1.46. The F:Ca atomic ratio was calculated as 0.167, slightly below the theoretical ratio of 0.2 for a fully fluoridated FHAp, suggesting only partial substitution of hydroxyl groups with fluoride. The reduced fluoride content compared to the hexagonal crystals suggests a lower substitution, which correlates with the observed morphological differences.

**Figure 3:**
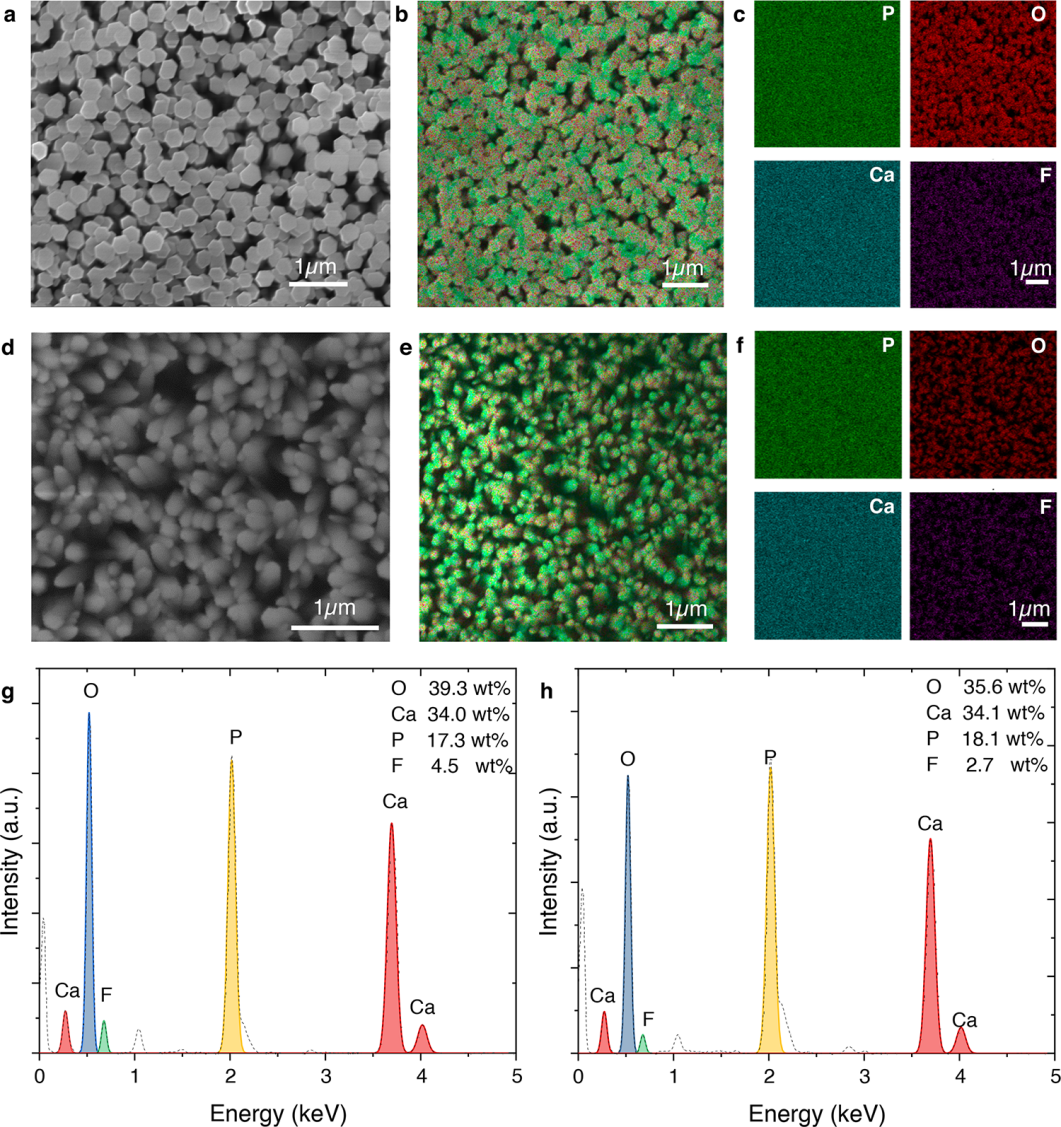
Tunable morphology of fluoridated hydroxyapatite at different F^–^ molar ratios. (a) SEM image of hexagonal FHAp crystals. (b) Energy-dispersive X-ray spectroscopy (EDS) merged elemental mapping of hexagonal FHAp. (c) EDS maps showing the distribution of P, O, Ca, and F in hexagonal FHAp. (d) SEM image of ovoid FHAp crystals. (e) EDS merged elemental mapping of ovoid FHAp. (f) EDS maps showing the distribution of P, O, Ca, and F in ovoid FHAp. (g) EDS analysis of hexagonal FHAp crystals synthesized with a fluoride concentration of 1.5 mM in the growth buffer. (h) EDS analysis of ovoid FHAp crystals synthesized with fluoride concentrations of 0.5 mM in the growth buffer.

There are two possible mechanisms that contribute to the observed morphological differences. (1) Hexagonal HAp crystals exhibit two primary crystal planes, indicated by their normal axis directions: the prismatic plane and the basal plane. Theoretically, the prismatic planes are enriched with calcium ions, rendering them positively charged, while the *c*-plane is predominantly composed of phosphate and hydroxyl ions, resulting in a more negatively charged facet.^41^ This anisotropic surface charge distribution imparts distinct properties to HAp, such as directional adsorption of molecules. Notably, during the synthesis of HAp crystals, the surface ionic composition and charge of HAp are not static but vary depending on the ionic composition of the surrounding aqueous medium due to ion exchange. (2) The anisotropic growth may also be influenced by the substitution of fluoride (F^–^) for hydroxyl ions (OH^–^) within the crystal lattice. This substitution plays a pivotal role in determining the morphology of hydroxyapatite or FHAp. Fluoride ions (F^–^) exhibit higher electronegativity than hydroxyl ions (OH^–^), primarily due to their smaller radius (71 pm for F^–^ versus 110 pm for OH^–^). This difference results in a stronger attraction of electrons with F^–^, giving it a formal charge of −0.95,^42^ whereas the formal charge of OH^-^ is slightly less negative at −0.9 due to the partial sharing of electrons within the hydroxyl group.^43^ Compared with hydroxyl ions, the assembly of fluoride tends to stabilize the crystal lattice, particularly along the *c*-axis, which enhances crystal packing and reduces structural defects. The substitution of fluoride also alters the surface charge distribution and ionic interactions, especially the packing of positively charged calcium ions in the *c*-plane, amplifying the anisotropic characteristics of FHAp. As a result, higher fluoride concentrations promote the development of a hexagonal crystal shape with well-defined facets, while lower fluoride concentrations result in ovoid-shaped nanostructures.

Nano-FTIR spectroscopy was employed in conjunction with a scattering-type scanning near-field optical microscope (s-SNOM) to achieve nanoscale chemical mapping of FHAp nanocrystals. This nanospectroscopic technique allows simultaneous morphological and nearfield spectral characterization with a spatial resolution of ∼20 nm, enabling direct correlation between local nanocrystal structure and infrared absorption properties.^44^ The typical near-field optical probing mechanism of s-SNOM relies on the nanoscale concentration of an illuminating field generated by a metallised AFM tip to yield a nanofocus. This nanofocus enables optical characterization at the spatial resolutions below the diffraction limit by detecting the scattered infrared light from the tip–sample interaction.^45^ To extract meaningful, background-free spectral and structural information, the detected scattered signal undergoes normalization against a silicon wafer reference, isolating the genuine near-field responses of the crystals. Figure 4a,d shows the representative topography and 3D surface morphology of hexagonal FHAp crystals, while the corresponding second-harmonic optical amplitude (O2A) map (Figure 4b) reveals the near-field IR absorption. The nano-FTIR spectra collected at the five consecutive scanning points (Figure 4c) suggest a homogeneous optical response of the hexagonal FHAp. The second-harmonic optical phase plots of O2P signal across the designated points display the characteristic phosphate vibrational modes (Figure 4e,f). Notably the *ν*_*1*_ symmetric stretching mode (∼967 cm^–1^) and *ν*_*3*_ asymmetric stretching mode (∼1064 cm^–1^) remain unchanged across all scans, further supporting the homogeneous optical and structural properties of the crystals. The additional bands at 1104.6 and 1146.8 cm^–1^, while observed consistently across all scan points, may correspond to perturbations in higher-order phosphate vibrational modes, potentially influenced by local structural distortions from fluoride substitution. The higher-frequency shift of the *ν*_*3*_ mode compared to commercial hydroxyapatite (∼1060 cm^–1^, Figure S3) may be attributed to lattice contraction due to fluoride substitution, which strengthens the phosphorus–oxygen (P–O) bonding.

**Figure 4:**
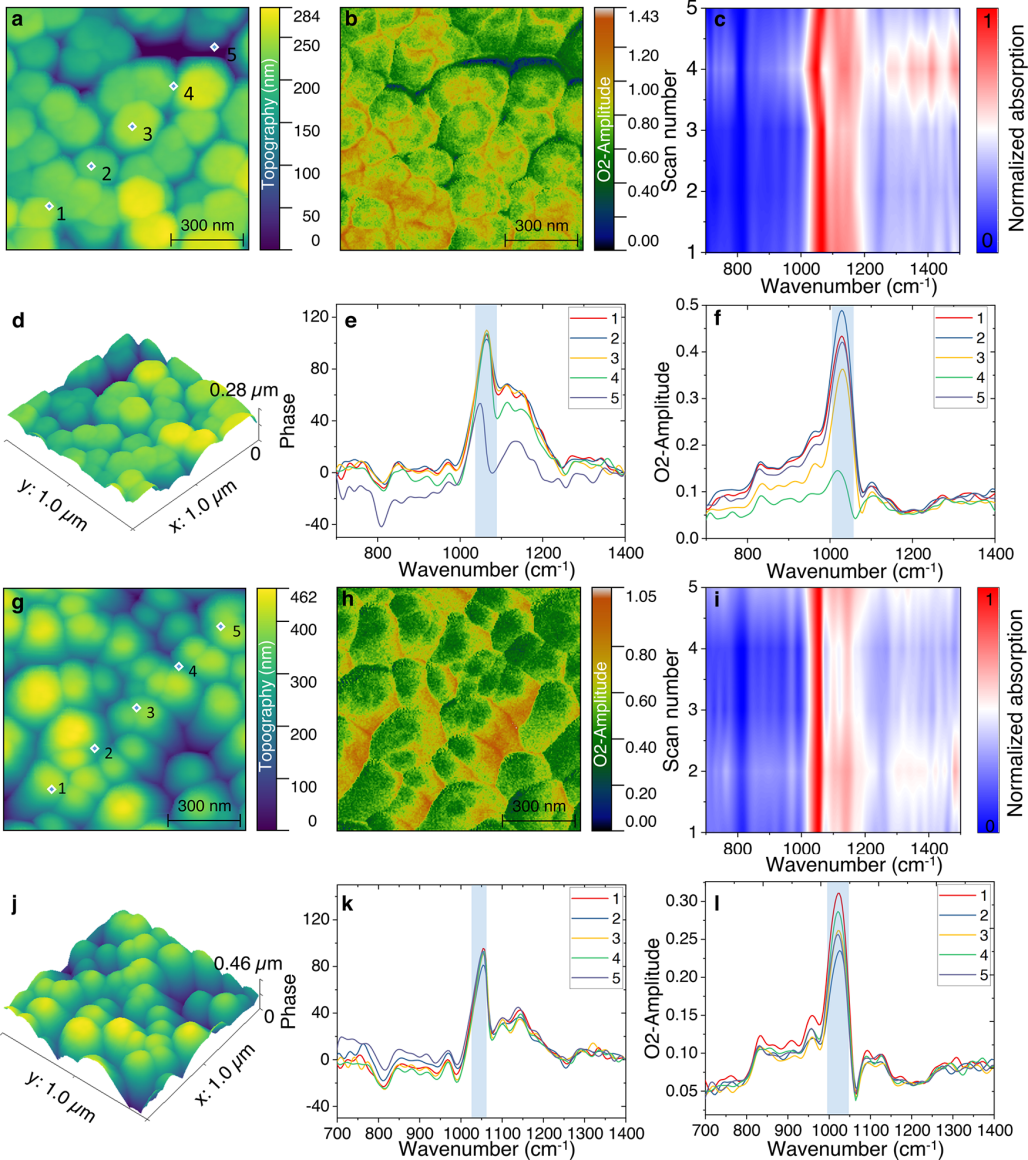
Nanostructure imaging of scattering-type scanning near-field optical microscopy and nano-Fourier transform infrared spectroscopy (nano-FTIR) of FHAp crystals. (a) Representative topography and (b) optical amplitude of hexagonal FHAp. (c) Corresponding local nano-FTIR absorption of scanning points in (a) (spatial resolution of ∼20 nm). (d) 3D height of measured hexagonal FHAp crystals. (e) Phase and (f) amplitude of the second-harmonic optical signal (O2P and O2A) measured at the highlighted points in panel (a). (g) Representative topography and (h) O2A optical amplitude of rod-like FHAp. (i) Corresponding local nano-FTIR absorption of the scanning points in panel (g). (j) 3D height of the measured rod-like FHAp crystals. (k) Optical phase and (l) amplitude taken across the highlighted points in panel (g).

In contrast, the ovoid FHAp crystals (Figure 4g) exhibit an elongated and less compact structure, as seen in the O2A amplitude map of IR absorption (Figure 4h). The nano-FTIR spectra (Figure 4i) show consistent optical responses across the crystal surface, further supporting a high degree of structural homogeneity, which is also confirmed by the 3D height topography (Figure 4j). The 2D plot of phase and amplitude in Figure 4k shows a primary *ν*_*3*_ asymmetric stretching mode at ∼1053 cm^–1^, along with ν_1_ at ∼967 cm^–1^. The ν_3_ mode shifts to a lower wavenumber (∼1053 cm^–1^) compared to hexagonal FHAp (1064 cm^–1^) and commercial HAp at ∼1060 cm^–1^, suggesting a weaker P–O bond environment, likely due to partial fluoride substitution, where residual hydroxyl groups remain in the lattice. Studies on calcium-phosphate materials have also identified distinct vibrational modes associated with the P–O bonding environments. In calcium-coordinated phosphate groups, the P–O stretching vibrations typically appear around 1025 cm^–1^, reflecting the influence of calcium interactions on the phosphate structure.^46–48^ In contrast, when phosphate groups are uncoordinated, as in densely packed calcium phosphate phases, the P–O valence vibrations shift to ∼1085 cm^–1^, indicating a more rigid and less perturbed bonding environment.^47^ Compared with the ATR-FTIR spectra in Figure S2, the *ν*_*3*_ asymmetric stretching mode in all measurements exhibits a red shift, with wavenumbers decreasing to approximately 1029 cm^–1^ for both HAp and FHAp. In contrast, the ν_1_ symmetric stretching mode remains unchanged at approximately 960 cm^–1^.

The interpretation of nearfield nano-FTIR spectra and their correlation with the far-field ATR-FTIR requires careful consideration of several scale-dependent aspects inherent to each of the technique in relation to the material nanoscale structures and properties. One limitation of scattering-type s-SNOM is the redistribution of vibrational band intensities, meaning that certain infrared absorption features may appear enhanced or suppressed relative to bulk spectra. This effect is often attributed to surface heterogeneity or the presence of nonapatite phases on individual nanocrystals, which can alter local dielectric environments and affect near-field signal contrast.^49^ This surface zone effect, coupled with compositional gradients within single crystallites, introduces complexity in distinguishing surface molecular contributions from bulk properties.^1,50^ Furthermore, the frequency shift in nano-FTIR spectra has been shown to depend on the surface probing depth, underscoring the need to account for the heterogeneity between surface and bulk regions. In enamel apatite, this compositional gradient is particularly pronounced, where surface calcium-phosphate clusters and nonstoichiometric substitutions may significantly influence the observed spectra.^51^ In light of this, nanoFTIR spectra are suited for the nanoscale characterization of the surface layers on crystals.

### 2.3 Mechanical and Chemical Stability of the Fluoridated Hydroxyapatite Nanocrystals

Structural differences in atomic bonding strength and atomic packing density across crystallographic directions lead to the anisotropic mechanical properties in single crystals. With a comprehensive understanding of chemical composition, molecular structure, and controlled orientation and morphology, the synthesized artificial enamel provides an excellent platform for investigating the anisotropic mechanical and chemical properties of HAp crystals. In FHAp, which crystallizes in a hexagonal structure, the *c*-plane (basal plane, {0001}, perpendicular to the *c*-axis) and the prismatic planes (prism side planes, such as {101̅0}, {011̅0}, or {112̅0}, exhibit distinct mechanical responses due to the differences in atomic arrangement and interatomic bonding forces.

To elucidate the anisotropic mechanical properties of FHAp, we conducted molecular dynamics (MD) uniaxial compression simulations (UCS) along both the *a*-axis and *c*-axis. A 12 × 12 × 4 unit cell was constructed to model the FHAp system. UCS simulations were performed to evaluate the effect of fluoride substitution on the mechanical anisotropy by applying uniaxial stress along the *c*-axis and *a*-axis until the material deformed into an amorphous phase, as illustrated in Figure S4a. The stress–strain curves were obtained from three independent simulations, and the Young’s modulus was calculated from the initial 10% linear region of the stress–strain response, averaging results from three separate calculations. The results in Figure S4a–g confirm the consistent presence of mechanical anisotropy across all fluoridated HAp systems, where the *c*-axis modulus remains significantly higher than the *a-*axis modulus. At 75% fluoride substitution, the *c*-axis Young’s modulus was determined to be 148.6 ± 0.88 GPa, compared to 140.01 ± 1.03 GPa along the *a*-axis, highlighting the directional dependence of mechanical stiffness. Interestingly, the fluoride concentration significantly influences the *c*-axis modulus, whereas the *a*-axis remains relatively stable. Compared to pure HAp, low to moderate fluoride substitution (≤75%) slightly reduces the *c*-axis modulus, while the *a*-axis remains largely unchanged. However, at full fluoride substitution (100%), Young’s modulus increases along both the *a-* and *c*-axis, suggesting enhanced lattice stiffness due to complete hydroxyl-to-fluoride replacement. It is worth noting that molecular dynamics calculations were performed on idealized single crystals, which may lead to overestimated mechanical properties compared to experimental results, where nano- and microscale crystals are more affected by boundary effects.

To validate the model-based predictions of anisotropic mechanical properties, nanoscale indentation by AFM was performed on the synthesized FHAp crystals. Unlike uniaxial compression (UCS), which applies uniform deformation along the *a*- or *c*-axis, indentation loading is confined to specific crystal facets, providing localized measurements of mechanical response. The anisotropic mechanical behavior under indentation is schematically illustrated in Figure 5a,d. AFM nanoindentation was performed on individual facets of the nanocrystals, assessing their mechanical response across different crystal planes. The samples were mounted using wax in two different orientations for AFM scanning, with the top surface and cross-section exposed to the scanning probe separately. AFM topography images (Figure 5b,e) provide nanoscale visualization of the FHAp surface. The hexagonal and rectangular facets are highlighted, confirming the distinct crystallographic orientations exposed to indentation.

**Figure 5:**
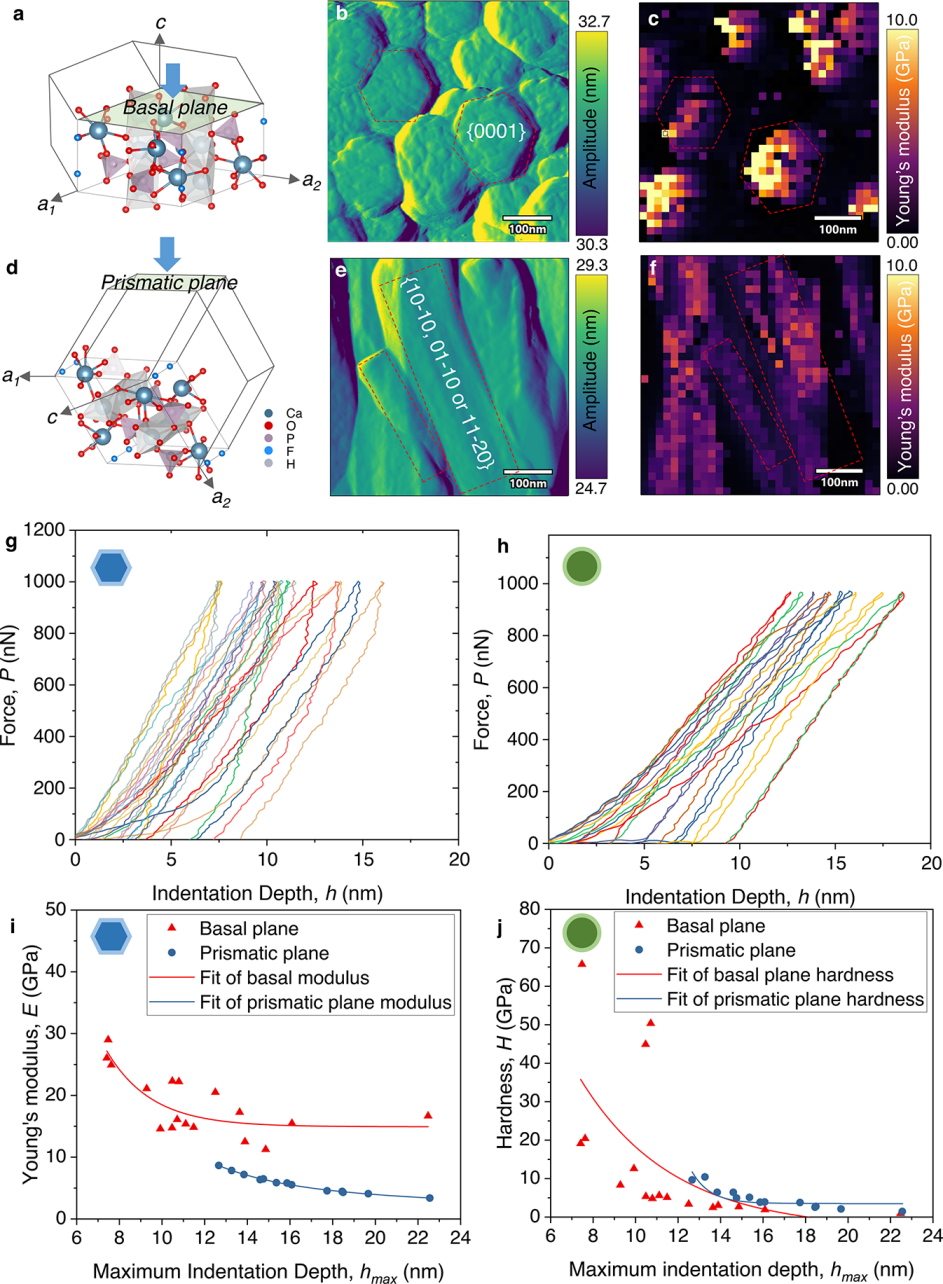
AFM nanoindentation of individual nanocrystals for anisotropic mechanical property determination at different crystal planes. (a) Crystal structure of hexagonal FHAp with the basal *c*-plane {0001} subjected to indentation study. (b) AFM amplitude topography of FHAp crystals with the *c*-plane exposed, acquired using a silicon tip (AC160 probe). (c) In situ indentation modulus mapping of the corresponding area in panel (b). (d) Crystal structure of hexagonal FHAp with the prismatic planes exposed to indentation. (e) AFM amplitude map of FHAp crystals with the prismatic plane exposed to indentation. (f) In situ indentation modulus mapping of the corresponding area in panel (e). (g) Representative force–displacement (*P–h*) curves from typical indentations in the basal *c*-plane indentation modulus mapping. (h) Representative force–displacement (*P–h*) curves from typical indentations in the prismatic plane indentation modulus mapping. (i) Young’s modulus (*E*) of the basal plane compared with the prismatic plane, calculated from the representative force–displacement curves, plotted as a function of maximum indentation depth (*h*_max_). (j) Hardness (*H*) of the basal plane compared with the prismatic plane, calculated from the representative force–displacement curves, plotted as a function of the maximum indentation depth (*h*_max_). Crystal structures of FHAp visualized using VESTA^52^ based on the CIF file from information card for entry 1010647 in the Crystallography Open Database.

For the nanoindentation experiments, an AC160 AFM probe was used as the indenting head, with a tip face angle (θ) of ∼35°, approximating a cube-corner indenter geometry. Indentation was performed in force-controlled mode, applying a maximum load force (*P*) of 1000 nN, corresponding to a surface penetration depth (*h*) between 5 and 20 nm. The indentation depth of all facets lies in the recommended 10% of the sample thickness, avoiding the substrate effects. We adopted the Oliver and Pharr (O&P)^53^ method to calculate the Young’s modulus (*E*) and hardness (*H*), which is applicable to the sharp-tip indenter geometry. In situ AFM indentation modulus maps were derived from an array of 32 × 32 indentations performed sequentially across the scanning area (Figure 5c,f), to evaluate the variation in mechanical properties across the investigated regions. The maximum modulus values are notably different between the basal plane and prismatic planes. The measured modulus values suggest that the basal *c*-plane possesses greater stiffness in certain regions, whereas the prismatic planes exhibit relatively higher compliance (hence low *E*). Figure 5g,h presents a set of representative indentation force–depth (*P–h*) curves obtained from indentation mapping. To ensure accurate calculation, precautions were taken to avoid slippage, collapse, and fracture manifestations in the loading curves,^54,55^ which mainly occur on side facets due to their orientation relative to the indentation direction. We further calculated Young’s modulus and hardness of the FHAp nanocrystals from each individual *P*–*h* curve based on the O&P method, using the equations given in the Supporting Information. The modulus and hardness obtained from 30 indentations are plotted in Figure 5i,j. The hexagonal FHAp crystals exhibited elastic anisotropy in the measured range, with an average elastic modulus of 18.4 ± 4.9 GPa, while the maximum elastic modulus (*E*_max_) reached ∼30.0 GPa and the minimum elastic modulus (*E*_min_) at 11.2 GPa on the *c*-plane (0001). In contrast, on the prismatic plane, the elastic modulus was significantly lower at 5.6 ± 1.4 GPa with *E*_max_ = ∼8.6 GPa and *E*_min_ = ∼3.3 GPa, indicating a pronounced direction-dependent mechanical behavior. Similarly, hardness measurements revealed substantial anisotropy. On the *c*-plane, the maximum hardness *H*_max_ = ∼65.7 GPa, while the *H*_min_ = ∼0.8 GPa. On the prismatic plane, the values were significantly reduced, with *H*_max_ = ∼10.3 GPa and *H*_min_ = ∼1.39 GPa, as shown in Table S1. These results highlight the strong crystallographic dependence of the mechanical response of FHAp, with the *c*-plane exhibiting a greater stiffness and hardness value compared to the prismatic planes. The nanoscale indentation modulus was further validated at the microscale using the iMicro indentation system, which measured the elastic modulus and hardness of the synthesized crystals along the *c*-axis within a 20 μm × 20 μm surface. The results, presented in Figure S5, demonstrate the uniformity of mechanical properties, with an average Young’s modulus of 31.36 ± 9.83 GPa (Figure S5a–c) and an average hardness of 1.63 ± 1.05 GPa (Figure S5d–f), further validating our nanoscale findings. However, larger-scale indentation mapping on the prismatic plane was not feasible due to the limited crystal length.

As illustrated in Figure 5a, the *c*-axis is aligned with the columnar arrangement of Ca^2+^ ions and phosphate tetrahedra (PO_4_^3–^), forming a stacked, tightly bound structure with strong ionic and covalent interactions. The hydroxyl (OH^–^) or substitution fluoride (F^–^) ions in FHAp are also oriented along the *c*-axis, further stabilizing the lattice. As a result, when indenting the (0001) plane, the deformation along this direction requires disrupting stronger Ca–O and P–O bonds acting like a stiff chain reinforcing the *c*-axis. In contrast, the prismatic planes contain more widely spaced Ca^2+^ and phosphate groups arranged in planar configurations, leading to weaker interlayer interactions and lower atomic packing density (Figure 5d). The mechanical response in these planes is dominated by weaker van der Waals forces and electrostatic interactions, making them more compliant and susceptible to deformation under mechanical stress.^56^ Additionally, the presence of lattice defects and local distortions in the prismatic planes may further lead to their relatively lower hardness and stiffness compared to the *c*-plane. It should also be noted that the mechanical properties measured at the nanoscale in this study are lower than those reported for large, well-faceted HAp crystals synthesized under high-temperature and high-pressure conditions—for example, ∼135 and ∼9.7 GPa for the (0001) basal plane in a crystal larger than 40 × 60 μm, as reported in ref 57. This discrepancy is primarily due to the smaller crystal size, lower synthesis temperature, which may include defects and additional grain boundaries. The nanoscale AFM indentation probes a localized volume, it is more sensitive to these heterogeneities. Thus, the presented combination of calculations and measurements should be taken to serve as qualitative indicators of crystallographic anisotropy, while accurate quantitative comparisons require large, defect-free crystals not representative of enamel-like structures.

A particularly valuable aspect of the present platform is its suitability for in situ analysis of demineralisation and remineralisation processes. Figure 6a presents sequential AFM three-dimensional height images capturing the dissolution dynamics of hexagonal FHAp when immersed in 10% lactic acid solution (pH 2.2) over 208 s. The progressive reduction in surface height suggests a layer-by-layer dissolution mechanism, as opposed to localized etching or pit formation. The initial maximum crystal height is approximately 290 nm, and the dissolution proceeds uniformly across the surface, with no evidence of preferential attack at grain boundaries or structural defects. Figure 6b provides a two-dimensional height map of the FHAp crystal. The time-lapse height changing profiles are extracted from the highlighted lines and reveal the topographic evolution during dissolution. Figure 6c presents the height profile along the *x*/*y* direction, quantitatively assessing the dissolution behavior within a local area. The profile demonstrates a consistent reduction in height across the scanned region, suggesting homogeneous dissolution. The absence of significant fluctuations or step-like features implies that surface dissolution occurs evenly rather than through localized dissolution pits or crystal cleavage steps. Figure 6d quantifies the evolution of height distribution over the 208-s dissolution period using the height distribution function (HDF). The HDF was calculated based on the normalized histograms of height values, providing a statistical representation of height evolution during dissolution. The average height decreases from 193.4 ± 35.47 to 157.5 ± 19.19 nm, corresponding to an average dissolution rate of 0.173 nm/s, which is equivalent to 15 μm per day, or 0.45 mm per month. This simple relationship highlights the importance of the proposed nanoscale platform for test acceleration, in that it enables rapid assessment of the rates of enamel dissolution and remineralisation on laboratory, rather than clinical time scales. The narrowing of the height distribution over time suggests that as dissolution progresses, the surface becomes increasingly uniform, likely due to the removal of loosely bound or structurally unstable regions.

**Figure 6:**
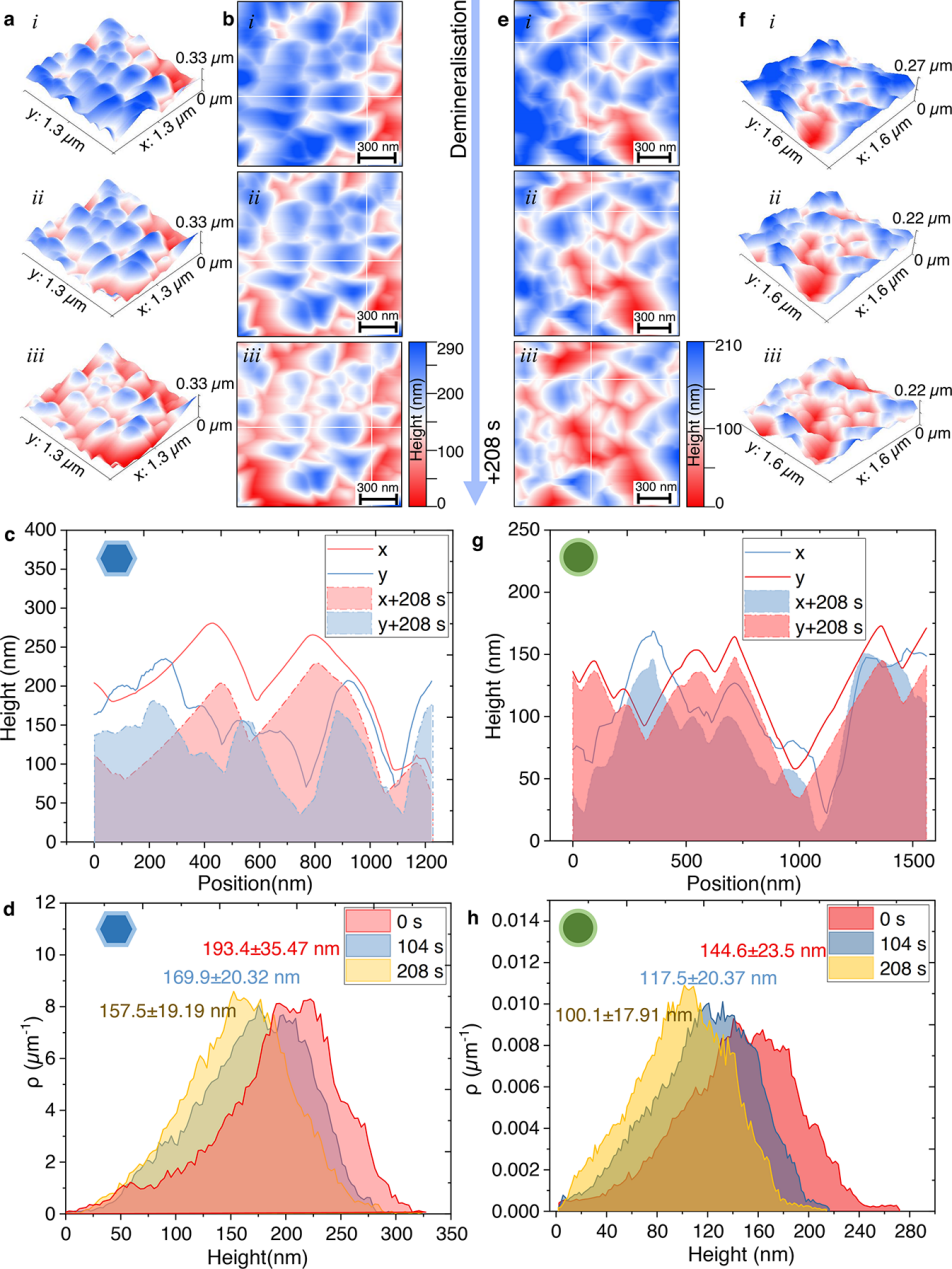
In situ AFM observation of hexagonal FHAp dissociation in lactic acid solution (10% volume) at pH 2.2 (a) 3D time lapse of AFM height images of hexagonal FHAp exposed to acid for 208 s. (b) 2D height image of the hexagonal FHAp. (c) Representative height profile of x/y direction extracted from (b). (d) Time-lapse height distribution quantification over 208 s of dissociation. (e) 2D time lapse of AFM height images of rod-like FHAp exposed to acid for 208 s. (f) 3D height image of FHAp in panel (e), highlighting the dissociation effect on surface height. (g) Representative height profile of *x*/*y* direction extracted from panel (e). (h) Time-lapse height distribution quantification over 208 s of dissociation.

Figure 6e–h provides further evidence of the FHAp dissolution behavior, focusing on the ovoid FHAp as a comparison to hexagonal FHAp. Previous studies have suggested that differences in crystal morphology could lead to significant variations in dissolution stability.^58^ The present results indicate that both hexagonal and ovoid FHAp exhibit a uniform dissolution process, without significant localization of degradation (Figure 6e–g). However, the slight difference in dissolution rates suggests an influence of fluoride substitution on structural stability. The height distribution analysis for ovoid-shaped crystals (Figure 6h) quantifies the reduction in average height from 144.6 ± 23.5 to 100.1 ± 17.91 nm over 208 s, corresponding to an average dissolution rate of 0.214 nm/s, equal to 18.5 μm per day, or in excess of 0.5 mm per month. This is almost 25% greater than the dissolution rate of hexagonal FHAp (0.173 nm/s, Figure 6d). The faster degradation of ovoid FHAp crystals may be attributed to the differences in fluoride substitution, as hexagonal FHAp incorporates a higher concentration of fluoride within the crystal lattice, whereas ovoid FHAp tends to have partial fluoride substitution, with residual hydroxyl (OH^–^) groups remaining. Since fluoride substitution strengthens P–O bonds and reduces lattice defects, hexagonal FHAp is expected to have greater acid resistance and chemical stability, which is consistent with its slightly lower dissolution rate.

Despite the difference in fluoride incorporation, the progressive narrowing of the height distribution over time suggests that both morphologies dissolve in a similarly uniform manner. Previous studies have reported that the dissolution of pure hydroxyapatite single crystals leads to pit formation on the basal plane, which serves as a preferential dissolution site^58^·^59^The absence of preferential facet etching or fissure-like degradation indicates that the dissolution process is structurally controlled, with fluoride incorporation playing a more dominant role than crystal shape alone in determining acid stability. Moreover, the AFM tip geometry used in this study may have influenced the observed topography. The FS1500 AFM probe, with a triangular tip shape (tip height: 3 nm, radius: 10 nm), introduces limitations in scanning deep features, potentially causing rounding artifacts in deep dissolution areas. This effect may partially obscure fine details of deep dissolution pits, leading to a smoothed surface representation. While this limitation does not alter the overall dissolution trend, it suggests that complementary high-resolution techniques, such as in situ transmission electron microscopy, could provide additional confirmation of the dissolution mechanism.^60^

FHAp was selected as a model system in this study, given its enhanced mechanical and chemical resistance, making it more stable under cariogenic conditions and pertinent to clinical products such as fluoride toothpastes. Its translational relevance is further supported by the fact that the surface of modern human enamel often incorporates fluoride through routine exposure. Structurally, the FHAp crystals synthesized in this study exhibit strong *c*-axis orientation, mimicking the alignment of natural enamel apatite along the enamel’s compressive axis. These features make FHAp a practical and biomimetic platform for *in situ* nanoscale studies. However, we acknowledge that FHAp does not fully replicate the hierarchical complexity of native enamel. Moreover, the experimental conditions do not encompass the full complexity of the oral environment, such as salivary flow, biofilm activity, and mechanical forces. Thus, while it serves as a useful model for fundamental mechanistic investigations, interpretations should be made with these limitations in mind.

## 3 Conclusions

This study introduces a facile synthesis method for fluoridated hydroxyapatite with precisely controlled crystal orientation and morphology, enabling detailed investigation of its structural, chemical, and mechanical properties with in situ and ex situ techniques. The synthesis approach can accommodate in situ AFM studies, providing a platform for real-time nanoscale observation of the mineralization dynamics. Chemical composition and molecular structure analysis confirmed that fluoride substitution in FHAp alters lattice parameters and allows exercising control over crystal morphology to create both hexagonal rod-like and ovoid crystals. Nearfield nanospectroscopic characterization revealed shifts in infrared vibrational modes, indicating reduced lattice disorder and well-packed crystalline structures in rod-like hexagonal FHAp crystals. These modifications contribute to slower solubility and improved acid resistance. The synthesized enamel-like crystals also enabled conducting tests for the assessment of anisotropic mechanical properties, revealing increased hardness and Young’s modulus along the *c*-axis. In situ AFM imaging further revealed a controlled dissolution mechanism, supporting FHAp’s enhanced resistance to acidic degradation. Overall, this work establishes an integrated experimental platform combining oriented FHAp synthesis, ex situ characterization, and in situ techniques to study nanoscale mineralization process. The findings bridge the gap from static *ex situ* analysis to real-time *in situ* observations conducted at the nanoscale, improving the fundamental understanding of the nanostructural, mechanical and chemical stability of FHAp and guiding the design of advanced biomimetic materials for enamel repair and protection. Future studies will focus on adapting this platform to native enamel substrates by developing local ion delivery strategies and surface preparation methods, paving the way for clinically translatable approaches to targeted enamel regeneration.

## 4 Materials and Methods

### 4.1 Methodology for Fluoridated Hydroxyapatite Crystal Synthesis

The synthesis of hydroxyapatite (HAp) crystals was performed using a carefully designed protocol to ensure the precise control of reactant concentrations and pH conditions. Amorphous calcium phosphate (ACP) powder was synthesized via precipitation from aqueous solutions. Reagent-grade CaCl_2_ and Na_2_HPO_4_ (Sigma-Aldrich) were separately dissolved in deionized (DI) water to prepare 10 mM solutions. Both solutions were chilled at 4 °C in an incubator before being rapidly mixed in a 1:1 volume ratio under continuous stirring for 1 min to induce precipitation. The resulting ACP precipitate was immediately filtered through a 0.22-μm cellulose acetate membrane and sequentially washed with 70% ethanol, followed by 99.5% ethanol to halt further reactions and remove residual moisture. The washed precipitate was then flash-frozen using liquid nitrogen for 30 min and subjected to vacuum drying for 24 h to ensure complete dehydration. The dried ACP powder was subsequently pellet-pressed into 9 mm discs under a 10-ton load for 5 min, producing compact samples for further use as substrates.

A phosphate buffer solution was prepared by dissolving 0.34836 g of potassium phosphate dibasic (K_2_HPO_4_) and 0.27218 g of potassium phosphate monobasic (KH_2_PO_4_) in approximately 800 mL of distilled water. To this solution, 4.1015 g of sodium acetate (CH_3_COONa) was added, and the mixture was stirred thoroughly until all components dissolved completely. The pH of the resulting buffer solution was adjusted to 6.3 by the gradual addition of 1 M acetic acid (CH_3_COOH) while monitoring the pH using a calibrated pH meter. The acid was added slowly to prevent overshooting the desired pH, with continuous stirring ensuring uniform adjustment.

Following the preparation of the phosphate buffer, 3.2 mL of 1 M calcium chloride (CaCl_2_) solution was introduced into the buffer and mixed thoroughly to achieve homogeneity. Subsequently, a sodium fluoride (NaF) solution with a concentration of 52.6 mM (1000 ppm) was prepared separately by dissolving 0.22087 g of NaF in distilled water to form a final volume of 100 mL. This NaF solution was added to the calcium- and phosphate-containing mixture in a volume ratio of 1:99 and 3:97, respectively, resulting in a final fluorine concentration of 0.526 mM (10 ppm) and 1.578 mM (30 ppm). The resulting growth buffer solution was used to grow HAp crystals by immersing substrates in the solution at 37 °C for varying durations, ranging from 30 min to 24 h. Postgrowth, the substrates were removed, rinsed with distilled water followed by an ethanol–water mixture of 70:30, and allowed to dry at room temperature. This procedure enabled the controlled synthesis of HAp crystals suitable for subsequent characterization and application.

### 4.2 AFM Imaging Methods

#### 4.2.1 Standard AFM Imaging in Air

SCOUT350 sharp silicon probe (NuNano) was used for high-resolution topographic imaging, with a tip radius of less than 5 nm, enabling the visualization of grain boundaries and fine structural details. The probe was operated in air tapping mode at a resonant frequency of ∼350 kHz with a standard spring constant of 42 N/m. AC160 TSA probe was used for standard AFM scanning, operating at 300 kHz with a spring constant of 26 N/m. The probe features a tetrahedral tip, ideally point-terminated, with a tip face angle of approximately 35°.

#### 4.2.2 In Situ Liquid Cell AFM Imaging of Remineralization

High-speed in situ atomic force microscopy imaging was conducted using an FS-1500AUD probe (Asylum Research), specifically designed for high-frequency imaging in liquid environments. The probe operates at a resonant frequency of approximately 1500 kHz with a standard spring constant of 6.0 N/m, ensuring high-resolution imaging during the remineralization process. To observe crystal growth in real-time, a growth buffer solution was prepared and loaded into a circulating injection system. The ACP substrate and AFM probe were initially immersed in ∼100 μL of deionized (DI) water and allowed to stabilize for 10 min until the scanning images reached equilibrium. The DI water was then gradually exchanged with the growth buffer solution while maintaining continuous scanning, ensuring a smooth transition. After 4–5 stable liquid exchange cycles, totalling 500 μL, the solution in the scanning region was considered fully replaced by the growth buffer, marking the initiation of crystal growth observation. Precautions: (1) ACP substrates exhibit some solubility in DI water, which can lead to partial dispersion of loose ACP particles. To minimize interference, the substrate was prewashed with DI water prior to imaging to remove loosely bound ACP, preventing turbidity in the observation solution. (2) Gas bubble formation was frequently observed during the crystallization process, potentially adhering to the probe and introducing additional liquid–gas interfaces, which could significantly affect imaging quality. To mitigate this, scanning was performed immediately after the buffer exchange to minimize bubble-induced artifacts.

#### 4.2.3 In Situ Liquid Cell AFM Imaging of Demineralization

For demineralization imaging, ACP substrates with overgrown crystalline layers were used in liquid-phase AFM tests. The sample and AFM probe were initially immersed in DI water for 10 min to allow stabilization. Subsequently, the DI water was replaced with 500 μL of lactic acid solution (10% v/v, pH 2.2) using five sequential exchange cycles in a circulating injection system, ensuring complete replacement of the liquid phase. The acid solution was prepared based on optimizations in previous research.^29,61,62^ Precautions: The crystalline top layer of the ACP substrate exhibited a porous nature, which could influence liquid exchange dynamics and dissolution behavior. Prior to immersing the AFM probe and sample in DI water, the sample surface was presoaked in DI water for ∼ 10 min to ensure complete surface wetting and removal of trapped air bubbles, preventing artifacts from gas–liquid interfaces during imaging.

### 4.3 Micro-/Nanoscale Indentation

#### 4.3.1 Nanoscale Indentation

Nanoscale indentation was performed using a Cypher AFM system (Oxford Instruments) equipped with an AC160 silicon probe, featuring a tetrahedral, ideally point-terminated tip with a tip face angle of approximately 35°. Samples were mounted separately with either the basal or prismatic planes exposed for indentation. The Oliver-Pharr method was used to calculate the elastic modulus from the unloading segment of the *P–h* curves, as detailed in the Supporting Information.

#### 4.3.2 Microscale Indentation

Microscale indentation was performed using an iMicro Nanoindenter (Nanomechanics Inc.) in NanoBlitz mode to assess the mechanical properties of the synthesized crystals on a microscale. The experiment was performed in a load control manner with a target load of 0.1 mN. A Berkovich diamond indenter tip was used to map a 20 μm × 20 μm area oriented normal to the *c*-axis, providing spatially resolved measurements of Young’s modulus and hardness. Oliver &Pharr method was used, and Poisson’s ratio of the sample was set to 0.33 in consistent with nanoscale indentation. The indentation array was designed to capture mechanical uniformity and anisotropy at microscale, thereby complementing the nanoscale indentation findings by AFM.

### 4.4 Structural and Compositional Characterization

#### 4.4.1 Scanning Electron Microscopy and Energy-Dispersive X-ray Spectroscopy

Surface morphology of the prepared hydroxyapatite samples was analyzed using a field-emission scanning electron microscope (FESEM, LYRA3 GM, TESCAN). The elemental composition of the samples was evaluated via energy-dispersive X-ray spectroscopy, conducted on the same microscope. The thickness of the upper crystalline layers was quantified based on SEM images using ImageJ software, based on measurements taken from 40 individual points at each time interval.

#### 4.4.2 X-ray Diffraction

The crystallinity and phase composition of the HAp samples were determined using X-ray diffraction (Rigaku MiniFlex) equipped with a Cu Kα radiation source λ = 1.541 Å.

#### 4.4.3 Fourier Transform Infrared Spectroscopy

Fourier transform infrared spectroscopy was performed using a Nicolet iS10 FTIR spectrometer equipped with an attenuated total reflectance (ATR) module to characterize the molecular structure and functional groups present in the HAp samples.

#### 4.4.4 NanoFTIR and Scattering-Type Scanning Near-Field Optical Microscope (s-SNOM)

The nanoscale chemical composition and local vibrational properties of the HAp samples were analyzed using nanoFTIR spectroscopy, conducted on an s-SNOM (Neaspec) coupled with a broadband infrared laser (Toptica Photonics AG). AFM was employed to obtain height topography images, using a platinum-coated Arrow NC-Pt probe (NanoAndMore GmbH) with a resonance frequency of 250 kHz and tip radius of ∼20 nm. Each nanoFTIR spectrum was obtained by averaging at least 20 Fourier-processed interferograms with a spectral resolution of 10 cm^–1^, recorded at 2048 points per interferogram with an integration time of 15 ms. The sample spectrum was normalized against a reference spectrum measured on a standard silicon surface, enabling the reconstruction of the second-harmonic optical amplitude and phase signals, denoted as O2A and O2P respectively.^44^ To extract the complex optical response of the material without background interference, the optical signal was demodulated at higher harmonics of the tip resonance frequency and processed using a pseudoheterodyne interferometric detection module, allowing precise measurement of amplitude and phase of the scattered wave.^63^

#### 4.4.5 Molecular Dynamics of Uniaxial Compression Simulations (UCS)

Fluoridated hydroxyapatite (FHAp) crystals were subjected to uniaxial compression simulations (UCS) to investigate their mechanical properties. The base HAp structure was obtained from the Interface Force Field (IFF) database,^43^ and fluoride substitution was introduced by partially replacing calcium ions with fluorine atoms using a custom Python script. A 12 × 12 × 4 supercell was constructed to model the FHAp lattice at varying fluorine concentrations. The system was equilibrated using the isothermal–isobaric ensemble (NPT ensemble) for 10 ns at 300 K and 1 bar, applying an anisotropic barostat to allow for direction-dependent volume fluctuations. Following equilibration, uniaxial compression was applied to the relaxed structure as detailed below. All molecular dynamics simulations were performed using LAMMPS (Large-scale Atomic/Molecular Massively Parallel Simulator), an open-source simulation package for atomistic modeling.^64^ Visualization and structural analysis were carried out using OVITO (Open Visualization Tool),^65^ while Python and Matplotlib were used to extract and plot the resulting stress–strain data.^66^

UCS were performed to examine the mechanical anisotropy of FHAp under varying fluoride substitution levels. Uniaxial compression was applied along the *c*-axis and *a*-axis until the structure collapsed into an amorphous state (Figure S4a). The stress–strain response was derived by averaging results from three independent simulations for each substitution level. The Young’s modulus (*E*) was calculated from the initial 10% linear portion of the stress–strain curve, ensuring consistency and accuracy in modulus estimation. The compression rate of 10^5^Å/s was adopted here.

## Supplementary Material





## Notes

Figure S1: XRD analysis of highly oriented FHAp crystals over 24 h; Figure S2: FTIR analysis and morphological comparison of fluoridated hydroxyapatite grown on a compressed ACP substrate; Figure S3: s-SNOM and nano-FTIR imaging of commercial HAp nanocrystals; Figure S4: uniaxial compression simulation results for HAp with varying fluoridation levels under anisotropic loading along the *a*- and *c*-axes; Table S1: AFM indentation data for Young’s modulus and hardness of anisotropic FHAp crystals; and Figure S5: microscale indentation mapping of hexagonal FHAp crystal along the *c*-axis (PDF)
Supporting Video 1: In situ LC-AFM observation of FHAp mineralization dynamics (MP4)
