# Supplementary material for: Biomimetic Enamel-like Crystals: A Versatile Platform for Unraveling the Basic Mechanisms of Demineralization and Remineralization
Source: ACS Appl Mater Interfaces. 2025 Aug 28;17(36):50505–18. doi: 10.1021/acsami.5c13544 (PMC12442002; doi:10.1021/acsami.5c13544)
Supplement: Supplementary file 1 [file am5c13544_si_001.pdf]

**Supporting Information**

# Biomimetic Enamel-Like Crystals: A Versatile Platform for Unravelling the Basic Mechanisms of Demineralisation and Remineralization

*Jinke Chang[1], Mahdi Tavakol[1], Cyril Besnard[1], Alexander M. Korsunsky[1**], and Jin-Chong Tan[1*]*

[1]Department of Engineering Science, University of Oxford, Oxford, Oxfordshire OX1 3PJ, United Kingdom

Corresponding Author:

**Alexander M. Korsunsky: korsunskygroup@gmail.com

* Jin-Chong Tan: jin-chong.tan@eng.ox.ac.uk

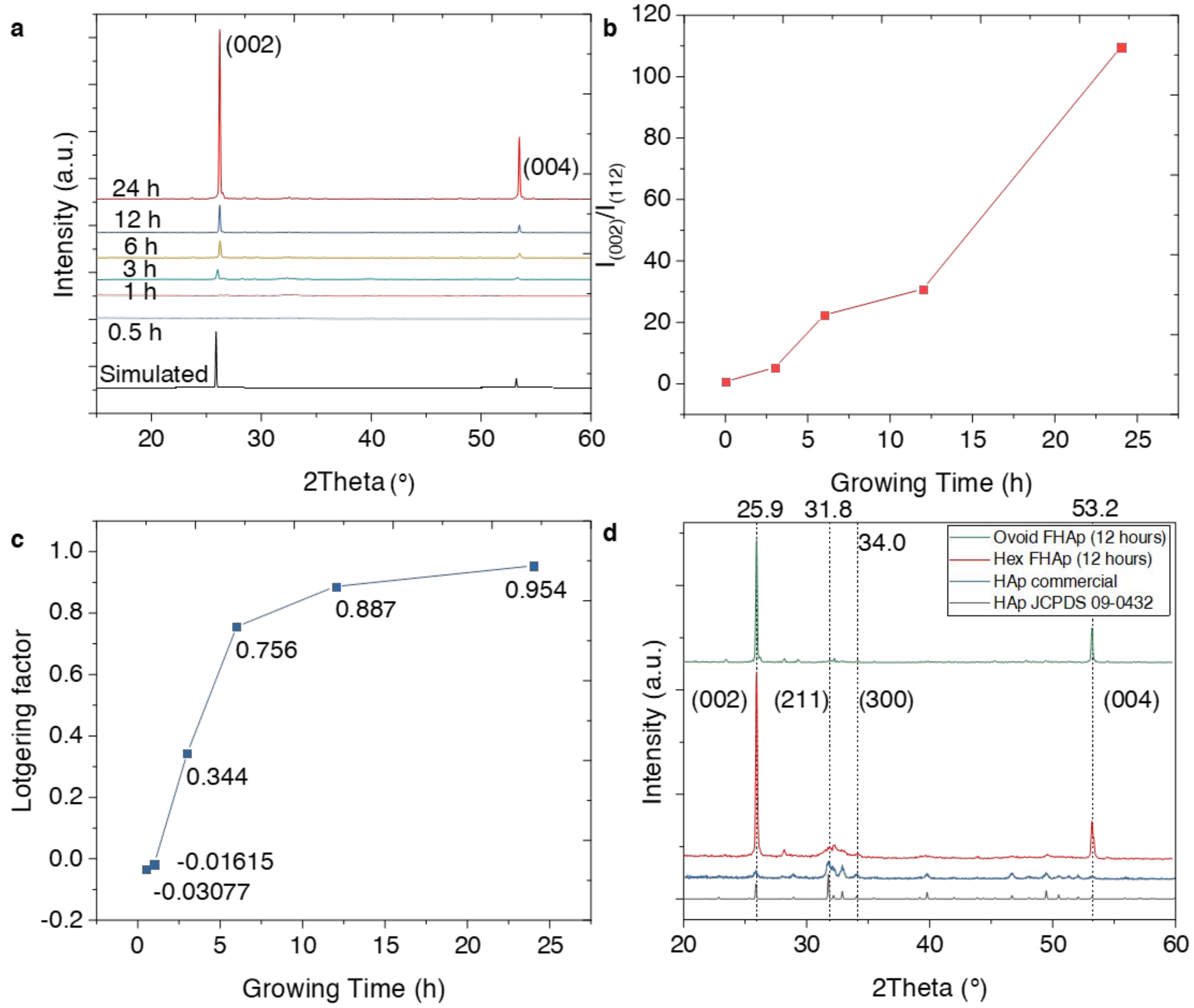


**Figure S1.** PXRD analysis of highly oriented crystals over 24 hours. (a) PXRD patterns of the synthesized ovoid-shaped crystals formed in 0.5 mM $F^-$ buffer solution for 24 hours, compared with the simulated PXRD pattern of a hydroxyapatite (HAp) crystal with a preferred *c*-axis alignment. (b) Intensity ratios of the (002) and (112) Bragg peaks of the crystals grown in solution. (c) Lotgering orientation factor of the synthesized crystals as a function of growth time. (d) Comparison of the PXRD patterns of the ovoid-shaped FHAp crystals (12-hour growth),

hexagonal-shaped FHAp, the commercial hydroxyapatite (HAp) nanoparticles, and the XRD reference pattern from JCPDS 09-0432.

The Lotgering orientation factor ($L$)[1] was determined to assess the preferred orientation and crystal shape of the overgrown layer using the PXRD peak intensity values. The factor was calculated using the following equation:

$$L = \frac{(P - P_0)}{(1 - P_0)}$$

where $P$ represents the ratio of the summed intensities of the ($00l$) reflections to the total intensities of all indexed reflections ($hkl$) for the sample:

$$P = \frac{\sum I(00l)}{\sum I(hkl)}$$

Similarly, $P_0$ corresponds to the same ratio derived from the standard hydroxyapatite reference pattern (JCPDS file 09-0432):

$$P_0 = \frac{\sum I_0(00l)}{\sum I_0(hkl)}$$

The Lotgering orientation factor quantitatively characterises the degree of preferential crystallographic alignment along the *c*-axis. A value approaching $L = 1$ signifies a nearly perfect orientation along the preferred direction, whereas a value approaching $L = 0$ indicates a randomly oriented polycrystalline structure.

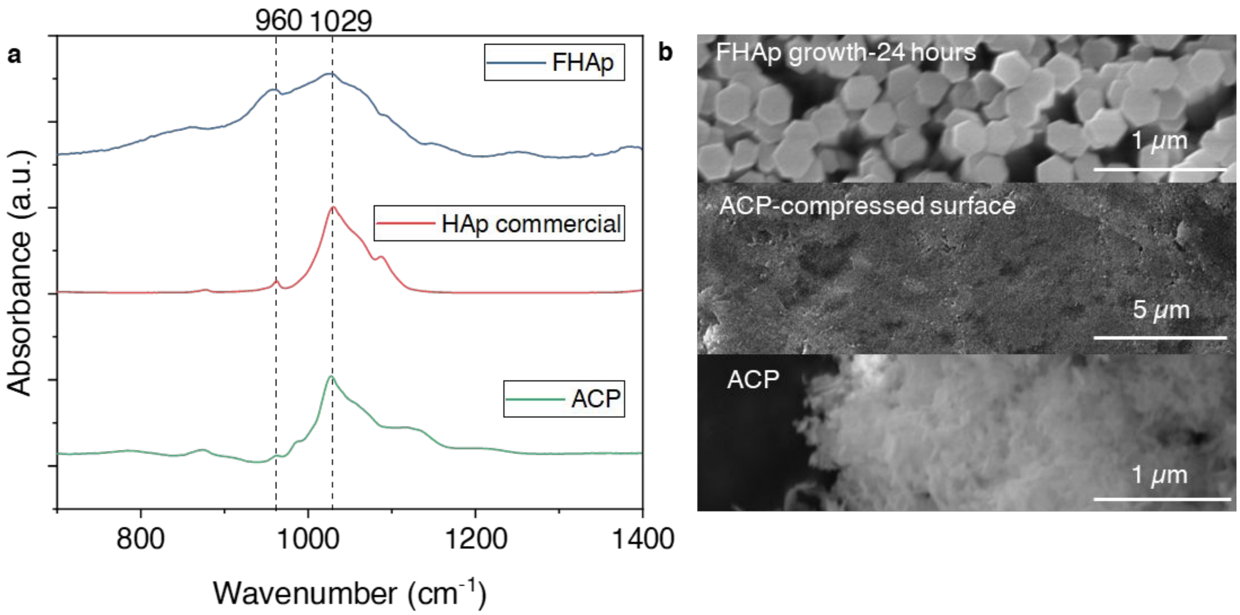


**Figure S2.** FTIR analysis and morphological comparison of fluoridated hydroxyapatite grown on a compressed ACP substrate. (a) Representative ATR-FTIR absorbance spectra and (b) typical SEM images showing the morphology of fluoridated hydroxyapatite, commercial HAp nanoparticles, and the initial ACP substrate.

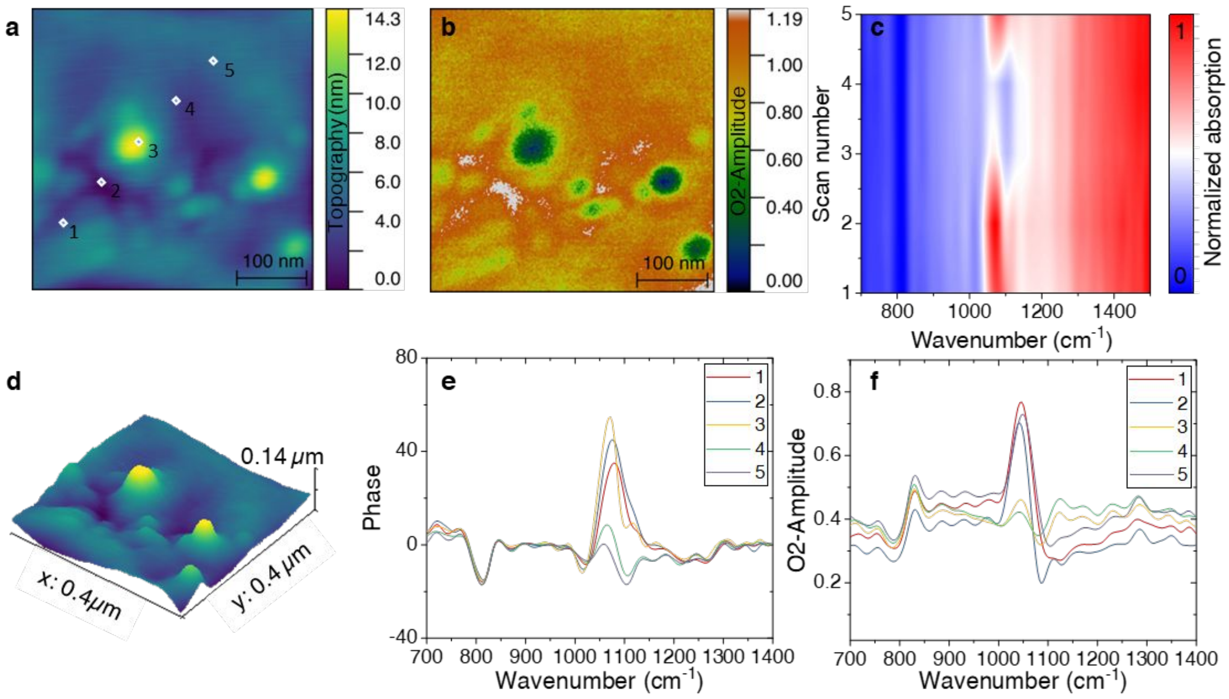

**Figure S3.** Nano-structure imaging of scattering-type scanning near-field optical microscopy and nano-Fourier transform infrared spectroscopy of commercial HAp nanocrystals. (a) Representative topography and (b) nearfield second-harmonic amplitude (O2A) of commercial nanocrystals, with (c) the corresponding local nanoFTIR absorption of scanning points in (a) (spatial resolution of ~20 nm). (d) 3D height topography of HAp nanocrystals. (e) Phase and (f) amplitude signals (O2P and O2A) of a line scan measured at the specific points marked in (a).

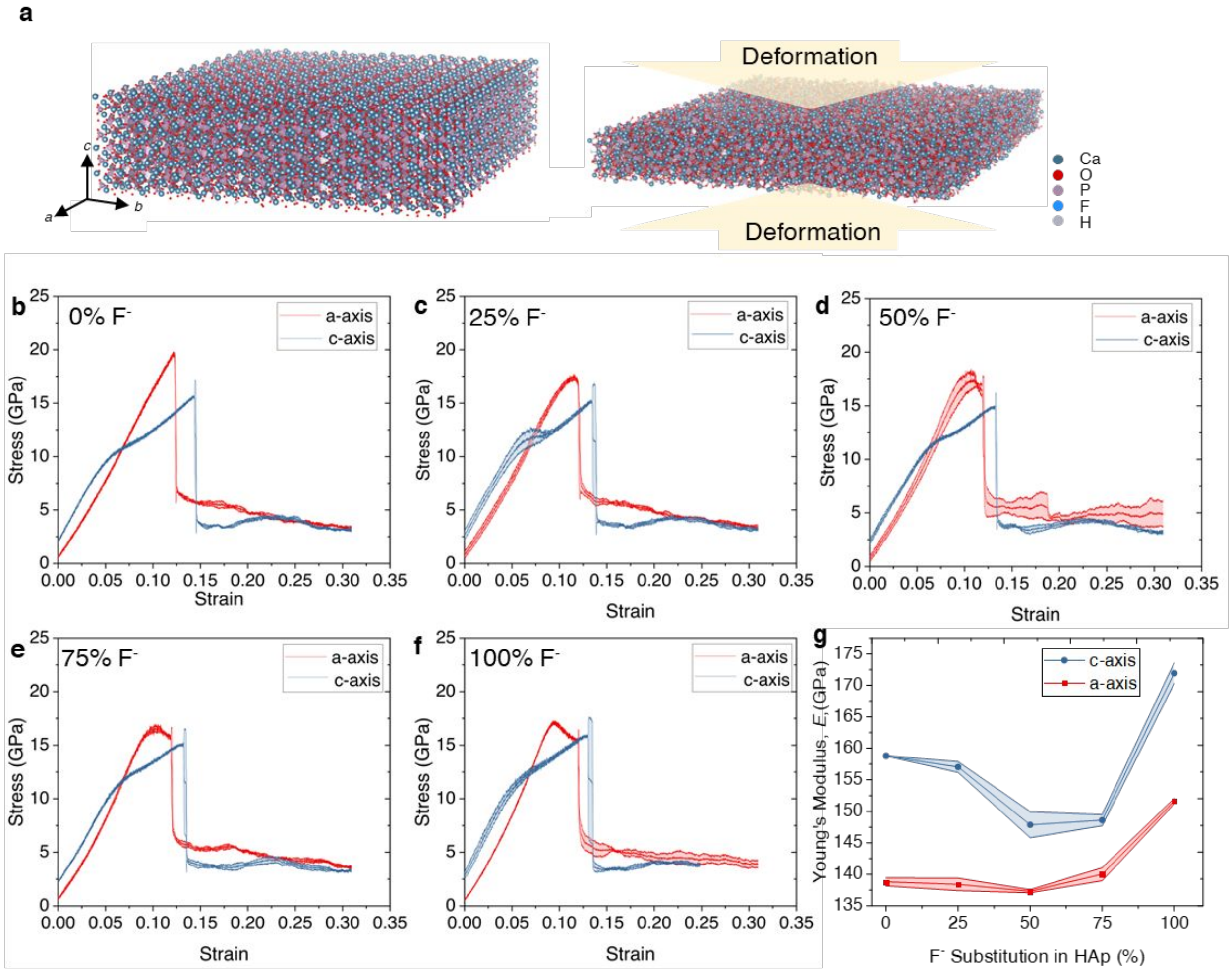


**Figure S4.** Uniaxial compression simulation results for HAp systems with varying fluoridation concentrations and anisotropic loading along the a-axis and c-axis. (a) Schematic illustration of

the simulation setup, showing the initial state (left) and the final state after deformation to the amorphous phase (right). Stress–strain curves for fluorine substitution at different atomic percentages: (b) 0%, (c) 25%, (d) 50%, (e) 75%, and (f) 100%. (g) Young's modulus derived from simulated compression loading along the a-axis and c-axis as a function of fluorine substitution.

**Table S1.** Young's modulus and hardness measured by AFM indentation of the anisotropic FHAp crystals. The data was derived from the indentation map in Figure 5, using the load-displacement curves, where the unloading test segments were analysed using the O&P method.

| Indentation number | *c*-plane | | *a(b)*-plane | |
|---|---|---|---|---|
| | Young's Modulus (GPa) | Hardness (MPa) | Young's Modulus (GPa) | Hardness (MPa) |
| 1 | 20.5 | 3274.2 | 8.6 | 9611.5 |
| 2 | 11.2 | 2571.6 | 5.8 | 5047.4 |
| 3 | 26.0 | 19034.5 | 5.5 | 3849.4 |
| 4 | 15.3 | 5516.3 | 7.8 | 10365.3 |
| 5 | 22.3 | 5273.7 | 6.3 | 6365.6 |
| 6 | 17.2 | 2404.9 | 4.4 | 2532.1 |
| 7 | 16.6 | 835.4 | 4.1 | 2042.7 |
| 8 | 12.5 | 2950.0 | 6.5 | 4897.5 |
| 9 | 29.0 | 65678.3 | 4.3 | 2702.5 |
| 10 | 14.6 | 12532.6 | 5.8 | 3790.9 |
| 11 | 24.9 | 20353.6 | 3.4 | 1384.3 |
| 12 | 21.1 | 8249.5 | 4.5 | 3712.1 |
| 13 | 22.2 | 4742.9 | 4.4 | 2484.3 |

| | | | | |
|---|---|---|---|---|
| 14 | 15.437 | 1825.4 | 7.151 | 6365.6 |
| 15 | 16.051 | 50318.8 | | |
| 16 | 14.700 | 44853.5 | | |
| 17 | 14.793 | 5026.1 | | |

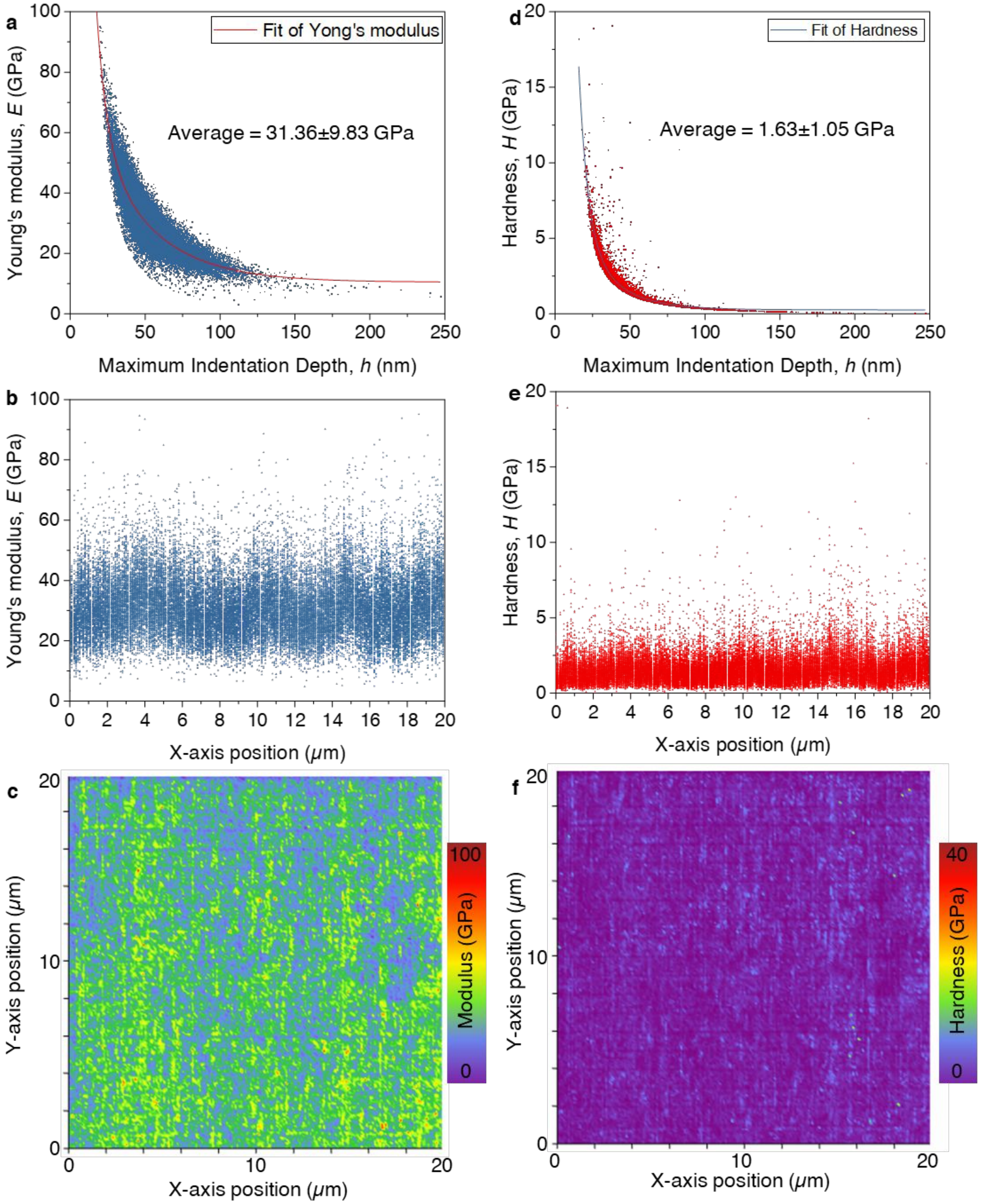


**Figure S5.** Microscale indentation of hexagonal FHAp crystal along the c-axis, conducted within a 20 μm × 20 μm scan window. (a) Young's modulus vs. maximum indentation depth, $h$. (b) Young's modulus distribution along the x-axis position. (c) 3D mapping of Young's modulus. (d)

Hardness vs. maximum indentation depth, $h$. (e) Hardness distribution along the x-axis position. (f) 3D mapping of surface hardness.

**Nanoindentation and Oliver-Pharr Method**

The Oliver-Pharr (O&P) method is the most widely used technique for extracting the mechanical stiffness (elastic modulus) from nanoindentation load-displacement ($P-h$) curves. Five key quantities are most important: the contact stiffness ($S$), contact area ($A_{contact}$), indentation hardness ($H$), reduced modulus $E_{reduced}$and the elastic modulus of the material ($E_{sample}$)[2].

The contact stiffness ($S$) is determined from the unloading segment of the $P-h$ curve as:

$$S = \frac{dP}{dh}$$

where $S$ is the contact stiffness (N/m), $P$ is the applied load (N), $h$ is the indentation depth (m). For a Berkovich indenter, the contact area is computed using:

$$\mathrm{A}_{contact} = \mathrm{C}_0 h_c{}^2 + \mathrm{C}_1 h_c{}^1 + \mathrm{C}_2 h_c{}^{1/2} + \mathrm{C}_3 h_c{}^{1/4} + \cdots$$

The first three terms were used for calculation, while higher-order terms were neglected due to their minimal contribution within the tested depth range. The AFM indenter can be defined as a cube-corner indenter, for which the expression for the projected area of contact is identical to a Berkovich indenter such that the angle $\theta$ is 35.26°, therefore

$$A_{contact} = 3\sqrt{3}\, h_c{}^2 \tan^2(\theta) = 2.60 h_c{}^2$$

$h_c$ is the contact depth which can be calculated as:

$$h_c = h_{max} - h_{sink-in} = h_{max} - \varepsilon \frac{P_{max}}{\left(\frac{dP}{dh}\right)}$$

where $\varepsilon \approx 0.75$ for a Berkovich indenter[3].

The indentation hardness can be calculated from the load and contact area:

$$H = \frac{P_{max}}{A_{contact}}$$

The reduced modulus is given by:

$$E_{reduced} = \frac{1}{2\beta} S \frac{\sqrt{\pi}}{\sqrt{A_{contact}}}$$

where $\beta$ is the dimensionless correction factor to account for non-symmetrical equivalence of the cube-corner indenter[4]. The Poisson's ratio of FHAp was taken as $\nu_{sample} = 0.33$, while for the indenter probe $\nu_{indenter} = 0.17$, thus $\beta$ was obtained as 1.0500.

The elastic modulus of the indented material ($E_{sample}$) is obtained from:

$$\frac{1}{E_{reduced}} = \frac{1 - \nu_{sample}{}^2}{E_{sample}} + \frac{1 - \nu_{indenter}{}^2}{E_{indenter}}$$

where $E_{indenter}$ is the elastic modulus of the indenter, while for this study $E_{indenter}$ = 150 GPa. The elastic modulus of the sample can be rearranged as:

$$E_{sample} = \frac{1 - \nu_{sample}{}^2}{\frac{1}{E_{reduced}} - \frac{1 - \nu_{indenter}{}^2}{E_{indenter}}}$$

## References


1. Lotgering, F., Topotactical reactions with ferrimagnetic oxides having hexagonal crystal structures—II. *Journal of Inorganic and Nuclear Chemistry* **1960,** *16* (1-2), 100-108.
2. Fischer-Cripps, A. C., *Nanoindentation, 2nd ed.* Springer: New York, , 2004; p XXII, 264.
3. Sneddon, I. N. J. I. j. o. e. s., The relation between load and penetration in the axisymmetric Boussinesq problem for a punch of arbitrary profile. *International journal of engineering science* **1965,** *3* (1), 47-57.

4. Hay, J. C.; Bolshakov, A.; Pharr, G. J. J. o. m. R., A critical examination of the fundamental relations used in the analysis of nanoindentation data. *Journal of materials Research* **1999,** *14* (6), 2296-2305.
